# Identification of a Novel Cis-Acting Regulator of HIV-1 Genome Packaging

**DOI:** 10.3390/ijms22073435

**Published:** 2021-03-26

**Authors:** Sayuri Sakuragi, Osamu Kotani, Masaru Yokoyama, Tatsuo Shioda, Hironori Sato, Jun-ichi Sakuragi

**Affiliations:** 1 Department of Viral Infections, Research Institute for Microbial Diseases, Osaka 565-0871, Japan; sakuragi-s@ncchd.go.jp (S.S.); shioda@biken.osaka-u.ac.jp (T.S.); 2 Department of Allergy and Clinical Immunology, National Center for Child Health and Development, Tokyo 157-8535, Japan; 3 Pathogen Genomics Center, National Institute for Infectious Diseases, Tokyo 208-0011, Japan; konioo@niid.go.jp (O.K.); yokoyama@nih.go.jp (M.Y.); 4 Division of Microbiology, Kanagawa Prefectural Institute of Public Health, Kanagawa 253-0087, Japan

**Keywords:** HIV-1, psi RNA, structure and function, genome packaging, RNA dimerization, structural dynamics, RNA fold, hydrogen-bond networks, molecular dynamics simulation

## Abstract

Human immunodeficiency virus type 1 (HIV-1) uptakes homo-dimerized viral RNA genome into its own particle. A cis-acting viral RNA segment responsible for this event, termed *packaging signal* (psi), is located at the 5′-end of the viral genome. Although the psi segment exhibits nucleotide variation in nature, its effects on the psi function largely remain unknown. Here we show that a psi sequence from an HIV-1 regional variant, subtype D, has a lower packaging ability compared with that from another regional variant, HIV-1 subtype B, despite maintaining similar genome dimerization activities. A series of molecular genetic investigations narrowed down the responsible element of the selective attenuation to the two sequential nucleotides at positions 226 and 227 in the psi segment. Molecular dynamics simulations predicted that the dinucleotide substitution alters structural dynamics, fold, and hydrogen-bond networks primarily of the psi-SL2 element that contains the binding interface of viral nucleocapsid protein for the genome packaging. In contrast, such structural changes were minimal within the SL1 element involved in genome dimerization. These results suggest that the psi 226/227 dinucleotide pair functions as a cis-acting regulator to control the psi structure to selectively tune the efficiency of packaging, but not dimerization of highly variable HIV-1 genomes.

## 1. Introduction

Retroviruses uptake homo-dimerized viral RNA genome into their own particle through an event that is called genome dimerization and packaging. It is an essential and highly-distinguished step for retroviral replication, as the ultimate objective for the virus is the conservation and propagation of its genome. The intrinsic structure on the 5′ end of the retroviral RNA genome is called the *packaging signal* (psi) and is recognized by the viral structural protein Gag [1]. A specific interaction between the psi segment and Gag is essential for accurate and efficient genome dimerization and packaging event, although its precise structures and machineries in changing cellular environments still remain unveiled.

Human immunodeficiency virus type 1 (HIV-1) belongs to Orthoretrovirinae, a subfamily of the retroviruses, and consists of genetically highly divergent strains in nature. They are primarily classified into subtypes in accordance with the homology of a genomic sequence, and, in addition, there is also a vast emergence of genetic recombinants, termed circulating recombinant forms (CRFs) [2,3]. More than ten subtypes and a hundred CRFs are reported thus far and are increasing in number year by year [4]. The genetic variation found in HIV-1 is thought to originate through two main mechanisms. The first mechanism is the frequent mutations introduced during the process of reverse transcription, which is caused by an error-prone viral reverse transcriptase (RT) [5,6]. The second mechanism involves the genetic recombination events that occur during reverse transcription. The CRFs occur as the result of the recombination event between the dimerized RNA genome during reverse transcription [7,8]. If there is an individual that is co-infected with multiple HIV-1 subtypes, a superinfected cell that harbors multiple subtypes could hypothetically emerge. Such a cell will likely produce an inter-subtype recombinant virus, which may serve as a potential ancestor of CRFs.

Previously we studied inter-subtype genome packaging and dimerization efficiency and reported the importance of stem-loop 1 (SL1) within the psi segment [9]. At the same time, we noticed that inter-subtype packaging of subtype D psi-containing genome into subtype B particle was inefficient, although the SL1 sequences of the two subtypes are phylogenetically similar among HIV-1 subtypes. To clarify this discrepancy, we investigated genetic determinants that generate the difference in viral genome packaging efficiency between these two subtypes. We determined the responsible element behind the packaging attenuation and further pursued its structural role through computer modeling/simulations.

## 2. Results

### 2.1. Packaging Inefficiency of Subtype D Psi

**Figure 1:**
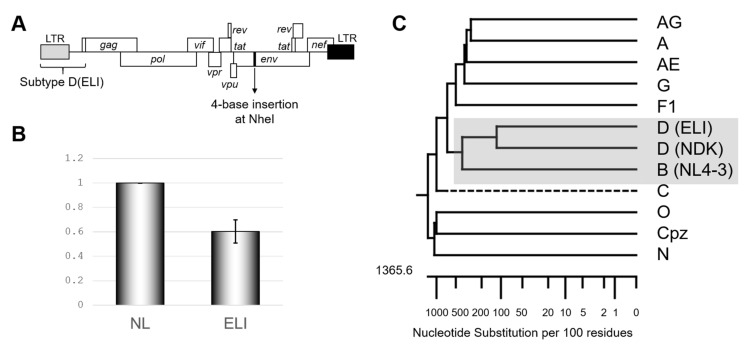
Confirmation of subtype D psi character. (**A**) Schematic of the backbone of the mutants examined in this study. (**B**) The genome package efficiency of the HIV-1 ELI (GenBank accession number: K03454) compared to NL4-3 (GenBank accession number: AF324493). The average of five independent experiments is shown. The error bar represents SEM. (**C**) Phylogenetic tree concerning packaging signal (psi) region of various HIV-1 subtypes, including SIVcpz. Drawn by MegAlign application in DNASTAR software (DNASTAR Inc., Madison, USA).

We first re-confirmed the differences in the psi-mediated genome packaging activities using an HIV-1 subtype B infectious molecular clone NL4-3 [10] and its recombinant possessing psi segment of the HIV-1 subtype D infectious molecular clone ELI (GenBank accession number: K03454) (Figure 1A). In our previous report, we measured packaging efficiency by comparing northern blot signal intensity per virion Gag amount [9]. This time we applied an alternative method, a competitive packaging assay utilizing RT-qPCR for quantification for viral RNA quantification, which provided a reliable quantitative value. The result obtained (Figure 1B) was consistent with that in the previous one [9] and suggested that subtype D psi has low packaging ability with subtype B virion. Notably, the psi elements from the subtypes B and D are most closely related in the genetic distance among HIV-1 subtypes (Figure 1C). As a quantifiable difference in the genome packaging activities was not detected with more distantly related psi(s) from other subtypes [9], this finding was rather unexpected.

**Figure 2:**
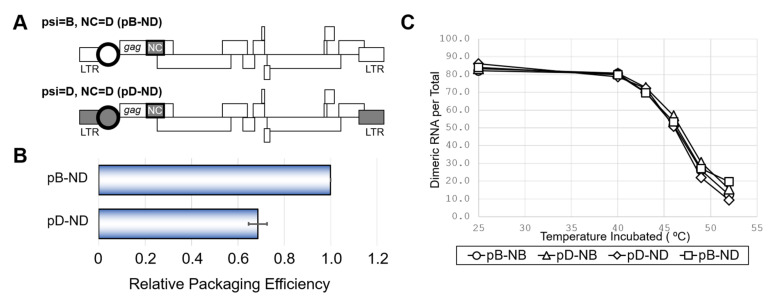
The effect of NC from subtype D on viral RNA packaging, dimerization and viability. (**A**) Schematic of the mutants obtaining NC of subtype D (ND) and accordant or discordant psi. (**B**) Packaging efficiency of the mutants. The average of three independent experiments are shown. Error bar represents SEM. (**C**) Dimerization ability of viral RNA from mutants with various combination of NC and psi. The representative data from at least three independent experiments are shown in C.

Because the HIV-1 genome packaging involves interactions between the psi segment and the viral nucleocapsid protein (NC) [1], we examined the effects of swapping the NC coding regions between the NL4-3 and ELI clones. In addition to the NL4-3 wild-type (pB-NB) and the mutant having ELI psi segment on the NL4-3 genetic backbone (pD-NB) (Figure 1A), two novel mutants carrying NC of ELI on the platform of pB-NB and pD-NB were constructed (pB-ND and pD-ND) (Figure 2A). As shown in Figure 2B, the packaging ability of pD-ND was lower than that of pB-ND, indicating the attenuation of subtype D psi element-mediated genome packaging activity is independent of NC protein subtype identity. HIV-1 genome packaging is tightly associated with viral genome dimerization [1]. However, the genome dimerization efficiency was not affected by the substitution of the psi/NC (Figure 2C), indicating that the differences in packaging abilities between subtypes were not due to differences in genome dimerization.

### 2.2. Precise Mapping of Determinant Region Affecting Packaging

**Figure 3:**
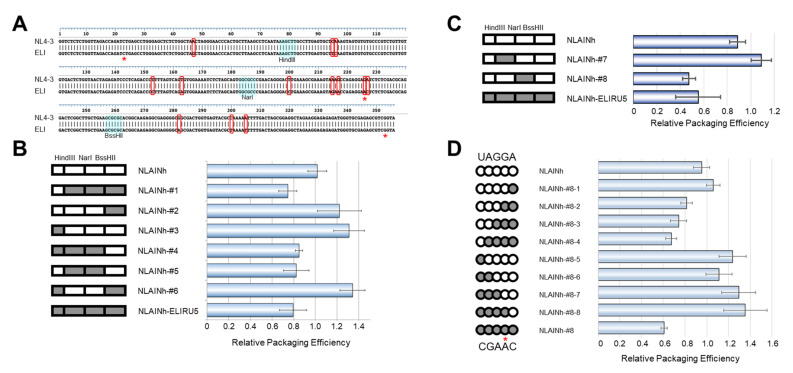
Genetic determinant of the subtype D psi phenotype. (**A**) Alignment of NL4-3 [10] and ELI strain psi sequences used in the experiment. The bases enclosed by red rectangles are discordant bases. The restriction enzymes applied for generating recombinants are shown. (**B**) First screening of the determinant. On the left, schematics of recombinant psi are shown. The parts from subtype B are white, and that from subtype D is gray. The data are the average of three independent experiments. Error bars represent SEM. (**C**) Second screening of the determinant. (**D**) Investigation of the base substitution mutants concerning five discordant bases (UAGGA/CGAAC) in NarI-BssHII fragment. (**B–D**) On the left, schematics of recombinant psi are shown. The parts from subtype B are white, and from subtype D are gray. The data are the average of three independent experiments. Error bars represent SEM. The red asterisks (*) on Figure 3A,D indicate the bases in our cloned ELI fragment, which are not identical with that of the ELI strain sequence registered in GenBank.

To pursue the determinant of this defect, we compared the nucleotide sequences of psi segments between the two subtypes. There are 13 differing nucleotides in this region examined between NL4-3 and ELI strains (Figure 3A). We generated several chimeric constructs utilizing common restriction enzyme sites within the psi segment and compared their genomic packaging efficiencies. After comparing these seven chimeras, mutant #5 was found to contain the minimal sequence responsible for the subtype D phenotype (Figure 3B, NLAINh-#5). Based on mutant #5, a second set of chimeras were constructed and examined (Figure 3C). Consequently, we identified mutant #8 to contain the minimal sequence required to retain subtype D packaging characteristics (Figure 3C, NLAINh-#8). Within the region, there are only five nucleotides differing between the two subtypes. We performed a base-substitution mutational analysis targeting these five nucleotides. Eight mutants were constructed to pursue the determinant of packaging efficiency, and we found that the consecutive nucleotide substitution from GA to AC at positions 226 and 227 of the HIV-1 NL4-3 [10] was responsible for the subtype D phenotype (Figure 3D). In addition, neither single-base changes lead to phenotype conversion while retaining subtype B packaging characteristics.

### 2.3. Location of the 226/227 Dinucleotide Pair in the Higher-Order Structure

**Figure 4:**
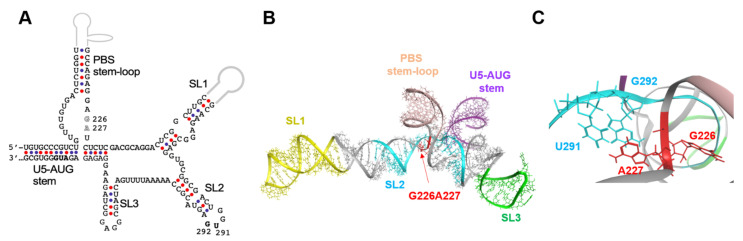
Location of the 226/227 dinucleotide pair in the higher-order structure. (**A**) Secondary structure of Ψ^CES^ [12], a minimal element for dimerization, NC binding, and packaging of HIV-1 RNA, are shown. Nucleotide sequence of the NL4-3 Ψ^CES^ [10], which is identical to that reported for the solution NMR structure of HIV-1 Ψ^CES^ (PDB code: 2N1Q) [11], was used to construct the secondary structure. Arrows indicate the 226/227 dinucleotide analyzed in this study. Arrow heads indicate a dinucleotide at positions 291 and 292, which were placed near the 226/227 dinucleotides in the three-dimensional structure. (**B**) Three-dimensional structure of the HIV-1 NL4-3 Ψ^CES^ [11] used for molecular dynamics (MD) simulations. The 226/227 dinucleotide is marked by red. Structural elements of the Ψ^CES^ were highlighted by different colors: U5-AUG stem (purple: positions 106–115 and 334–343), SL1 (yellow: positions 243–254 and 264–277), SL2 (cyan: 282–300), SL3 (green: 312–325), and PBS stem-loop (skin: 125–131 and 217–223). (**C**) Enlarged view of the Ψ^CES^ region around the G226/A227 dinucleotide.

To address the possible structural roles of the 226/227 dinucleotide in the psi function, we first analyzed the 3D location of the 226/227 dinucleotide within the psi segment using a ligand-free NMR structure of the “core encapsidation signal Ψ^CES^” [11]. The Ψ^CES^ consists of about ~160 nucleotides and covers the minimal sequence required for proper RNA dimerization, NC binding, and packaging [12] (Figure 4A). The NL4-3 Ψ^CES^ adopted a unique architecture, termed the *tandem three-way junction* [11], forming tetrapod-like architecture (Figure 4B). In the 3D structure of the NL4-3 Ψ^CES^, the dinucleotide at positions 226 and 227 in the base of the PBS stem-loop region was placed adjacent to the three-way junction area (Figure 4B). The dinucleotide was also placed near the dinucleotide at positions 291 and 292 in the SL2 loop tip (Figure 4C), which are located away from the 226/227 dinucleotide in the secondary structure (Figure 4A).

### 2.4. Effects of the Dinucleotide Substitution on the Structural Dynamics of The Psi

**Figure 5:**
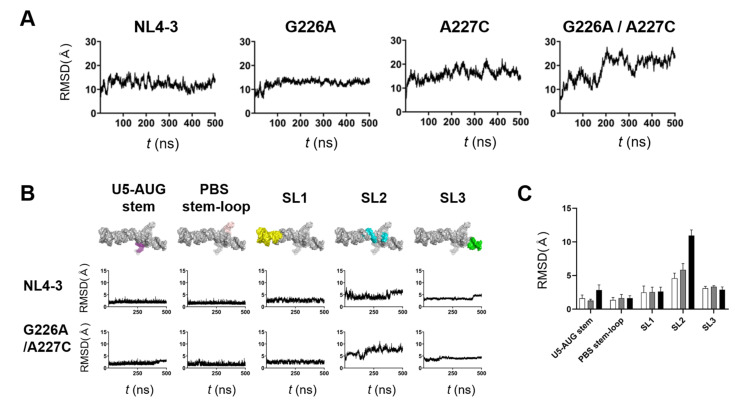
Effects of the dinucleotide substitution on the structural dynamics of psi. Ψ^CES^ models without or with indicated substitution were subjected to MD simulations using the Amber 16 program package [19]. Structural changes during 0–500 ns of MD simulations were monitored with the root mean square deviations (RMSDs) between the initial model structure and the structures at given time points of MD simulation using the cpptraj module in AmberTools 16 as described previously [20,21,22,23,24]. (**A**) and (**B**) Changes in RMSDs during MD simulations. Ψ^CES^ RNAs without or with indicated substitution (**A**). Structural units of Ψ^CES^ RNAs without or the 226/227 dinucleotide substitution (**B**). (**C**) Means of RMSDs of indicated structural units of Ψ^CES^ RNA. Structural units of Ψ^CES^ were extracted from Ψ^CES^s at 460, 470, 480, 490, and 500ns after MD simulations, superposed, and used for calculating RMSDs between NL4-3 and mutants. White, gray, black bars indicate RMSDs between NL4-3 Ψ^CES^ and Ψ^CES^s with a G226A substitution, an A227C substitution, and a G226A/A227C dinucleotide substitution, respectively. Mean values and standard deviation with the five time points are shown.

The structural dynamics of protein and nucleic acids play key roles in molecular interactions and, thereby, their functions [13,14,15,16,17]. To address any possible impacts of the G226A/A227C dinucleotide substitution on the structural dynamics of the Ψ^CES^ RNA, we conducted molecular dynamics (MD) simulations of the NL4-3 Ψ^CES^ models with and without the substitution. The root-mean-square deviations (RMSD) between the initial structure and those at given time points of MD simulation sharply increased within a few nanoseconds (Figure 5A), suggesting a quick relief of the structural distortions of the initial models at 37° C under solvent conditions. The RMSDs had reached a near plateau after 350 ns of MD simulations, although Ψ^CES^ element with the dinucleotide substitution exhibited more extensive structural fluctuations as compared with Ψ^CES^ wild-type and Ψ^CES^ with a G226A or an A227C single substitution (Figure 5A).

To elucidate fluctuating portions of the Ψ^CES^ elements, the Ψ^CES^ RNA was divided into 5 structural elements, i.e., U5-AUG stem, PBS-stem-loop, SL1, stem-loop 2 (SL2), and stem-loop 3 (SL3), and sequential changes in the RMSDs were monitored using 250,000 structures (snapshots) obtained by the MD simulations. Interestingly, U5-AUG stem, PBS-stem-loop, SL1, and SL3 elements had retained the initial structure during MD simulations in Ψ^CES^ RNAs regardless of the dinucleotide substitution, as evidenced with the low levels of RMSDs and their fluctuations within a few to several angstroms (Figure 5B). In contrast, RMSDs were significantly greater with the SL2 element than the others independent of the dinucleotide substitution (Figure 5B, SL2), suggesting that the SL2 element intrinsically possess greater levels of structural flexibility in the Ψ^CES^ RNA than the other elements do. Notably, however, RMSDs were much greater with the SL2 element with the G226A/A227C dinucleotide substitution than that without the substitution (Figure 5B, SL2), suggesting that the dinucleotide substitution predominantly increases structural dynamics of SL2 in the Ψ^CES^ RNA at 37 °C in a solvent environment. Similarly, the effects of single substitutions on RMSDs were greatest in SL2, although they were smaller than that of dinucleotide substitution (Figure 5C). It should be noted that the psi SL2 contains the binding interface to the HIV-1 NC protein that assists in genome packaging [18].

### 2.5. Effects of the Dinucleotide Substitution on the Fold of Psi

**Figure 6:**
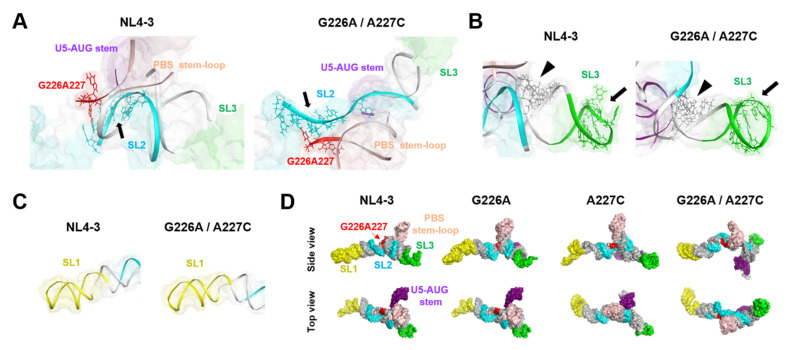
Effects of the dinucleotide substitution on the fold of psi. (**A**) Enlarged views around the three-way junction of the tetrapod-like structure of Ψ^CES^s at 500 ns of MD simulations. Arrows indicate an interface for the NL4-3 NC protein binding (positions 290–294 and 296 in SL2) (PDB code: 1F6U [18]). (**B**) Enlarged views around the SL3 structure of Ψ^CES^s at 500 ns of MD simulations. Arrows indicate an interface for the NL4-3 NC protein binding (positions 314–316 and 318–322 in SL3) (PDB code: 1A1T [25]) and arrow heads indicate an interface for the initial NC protein binding sites (positions 306–309 and 328–331 in three-way-junction) [26] (**C**) Enlarged views around the SL1 structure. (**D**) Comparison of overall structures of Ψ^CES^s at 500 ns of MD simulations. The Ψ^CES^ structures are viewed from the same direction after the superposition using AmberTools 16 [19].

The folding of biomolecules determines the exposure levels of interaction surfaces and thus plays key roles in molecular interactions and their functions. To address the effects of the G226A/A227C dinucleotide substitution on the conformation of the Ψ^CES^ RNA, the structures with and without the substitution at 500 ns of MD simulations were compared. The most remarkable change in the conformation was detected with the SL2 element for the NC-protein-binding [18]; the G226A/A227C dinucleotide substitution induced deformation of the SL2 from a bending curvature to an extended linear conformation (Figure 6A). On the other hand, the SL3, which contains another potential binding site for the NC protein [25,26], basically retained its initial conformation after the 226/227 dinucleotide substitution (Figure 6B). Likewise, the conformation of the SL1 stem, which forms an extended duplex for the initiation of genome dimerization [27,28,29], was not significantly affected by the dinucleotide substitution (Figure 6C). Due to the marked conformational changes in the SL2 element, the overall conformation of the Ψ^CES^ with the dinucleotide substitution changed from a tetrapod-like to a cross-like architecture (Figure 6D, G226A/A227C). Meanwhile, the conformation of the Ψ^CES^ with G226A or A227C single substitution was similar to that without substitution, basically maintaining the tetrapod-like architecture (Figure 6D, G226A and A227C).

### 2.6. Effects of the Dinucleotide Substitution on the Hydrogen-Bond Networks of the Psi

Intramolecular hydrogen bond networks play a key role in the stability of RNA structure and thus play key roles in molecular interactions and thereby their functions [30,31,32]. To examine whether the G226A/A227C dinucleotide substitution could affect intramolecular interactions of the Ψ^CES^ segment, we analyzed the hydrogen-bond networks of the Ψ^CES^ with and without the dinucleotide substitution. The hydrogen bonds associated with particular residues were extracted from 20,000 snapshots obtained with MD simulations between 300 and 500 ns of MD simulations, and levels of strength of the inter-residue connections were illustrated schematically with the thickness of links that directly correlate with the numbers of the hydrogen bonds formed between fluctuated nodes (residues) in the psi RNA during MD simulations.

**Figure 7:**
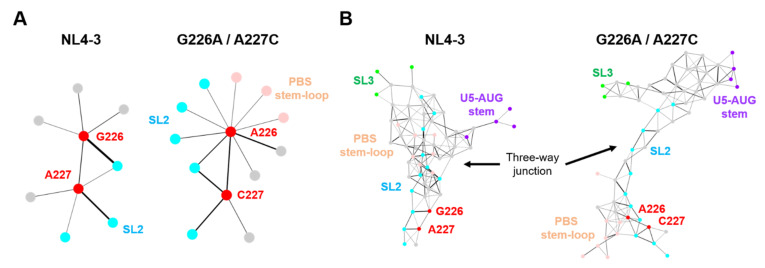
Effects of the dinucleotide substitution on the hydrogen-bond networks of psi. Hydrogen-bonds formed in the Ψ^CES^s were extracted from 20,000 trajectories obtained between 300 and 500 ns after the start of MD simulations. The hydrogen bonds associated with particular residues were extracted and visualized by Cytoscape platform [33], a software to analyze biomolecular interaction networks. (**A**) The hydrogen-bond networks of the dinucleotide at position 226 and 227. Circles indicate residues forming hydrogen bonds with the dinucleotide. Bar thickness correlates with the numbers of the hydrogen bonds formed in the fluctuated RNAs during MD simulations. (**B**) The hydrogen-bond networks around the three-way junction (nucleotide positions at 115–127, 222–227, 291–311, and 326–333).

Hydrogen bonds were formed between residues at positions 226 and 227 of the NL4-3 psi segment (Figure 7A, NL4-3), which could contribute to the stabilization of the 3D structure of the dinucleotide. In addition, these residues individually form several hydrogen bonds with surrounding residues. Interestingly, the 226/227 dinucleotide substitution on the NL4-3 Ψ^CES^ induced a remarkable change in the profile of the hydrogen-bonded base interactions at the substitution sites, creating multiple new edges between the residue at the 226 in the SL2 and those in the PBS stem-loop or the SL2 element (Figure 7A, G226A/A227C). In addition, hydrogen-bond formation between the dinucleotide was augmented by the dinucleotide substitution, as indicated by the thicker link connecting the dinucleotide nodes (Figure 7A, G226A/A227C). These changes in profiles of the base interactions were associated with substantial changes in the hydrogen-bond networks at the SL2 and three-way junction area (Figure 7B). Consistent with the experimental and in silico data (Figure 2C and Figure 6C), the hydrogen-bond networks in the SL1 stem, which is involved in genome dimerization, were preserved after the dinucleotide substitution (Figure S1).

### 2.7. Genetic Variations at the 226/227 Dinucleotide Pair and Its Flunking Regions

**Figure 8:**
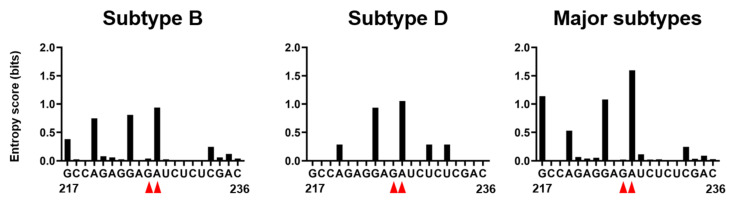
Nucleotide variations at the 226/227 dinucleotide pair and its flunking regions of HIV-1 psi segment. Shannon entropy scores representing variations at individual nucleotide positions were calculated using 815, 20, and 1556 sequences of HIV-1 subtype B, subtype D, and HIV-1 major subtypes, respectively [2,3] from different geographic regions in the world [34]. Nucleotide sequence of HIV-1 subtype B infectious molecular clone NL4-3 [10] is shown in horizontal axis along with corresponding Shannon entropy scores in y-axis. An entropy score of 0 indicates absolute conservation, whereas a score of 2.0 indicates complete randomness. Red arrow heads represent the dinucleotide at position 226 and 227.

Genetic variations at the 226/227 dinucleotide pair and its flanking regions were examined using the public HIV Sequence Database [34]. Shannon entropy scores [35] were used as the quantitative indicator of individual site variations; an entropy score of zero indicates absolute conservation, whereas a score of 2.0 bits indicates complete randomness. Scores obtained for the residue at position 226 were 0.038, 0, and 0.022 bits for the HIV-1 subtype B (*n* = 815), subtype D (*n* = 20), and HIV-1 major subtypes (*n* = 1556), respectively (Figure 8). The results indicate that the residue 226 is highly conserved within and among HIV-1 subtypes, as seen at functional sites of the viral enzyme [36] and structural protein [20]. In contrast, Shannon entropies for the 226 residues were much greater with the values of 0.937, 1.054, and 1.596 for subtype B, subtype D, and HIV-1 major subtypes, respectively (Figure 8). The results indicate that residue 227 is highly mutable in nature, suggesting that the residue is nonessential for the viral survival or the critical site for the successful viral adaptation in nature.

## 3. Discussion

Recent reports indicate the prevalence of a wide variety of HIV-1 CRFs all over the world [2,3]. The emergence of CRFs originates from the co-packaging of two different genomic subtypes into one virion. Thus, we were interested in and examined the compatibility of genome packaging and dimerization between various subtypes. In the current study, we provide in vitro and in silico evidence suggesting a molecular mechanism by which HIV-1 controls the compatibility. The obtained findings provide new insights into the regulation of structure and activity of the HIV-1 psi RNA by mutations and have interesting virological implications for HIV-1 adaptability.

Our previous [9] and present (Figure 1B) studies revealed that the psi RNA segment of the HIV-1 subtype D, which is predominantly prevalent in the African continent [2], did not function well in the viral genome packaging process when compared with that of subtype B, which has spread into all over the world [2]. Meanwhile, the subtype D psi segment functions well for the viral genome dimerization (Figure 2C), indicating that the effects of variation between subtype D and subtype B psi segments are rather specific to the genome-packaging. A series of molecular genetic studies successfully narrowed down the location of viral RNA sites for the subtype D-psi-dependent attenuation of viral genome packaging (Figure 3). The phenotypic change induced by the HIV-1 subtype D psi segment was not recovered by supplying NC protein from the HIV-1 subtype D (Figure 2B). The results suggest the attenuation of genome packaging efficiency of the subtype D psi segment is not due to the disharmony of cis- and trans-acting factors for the genome packaging.

There are thirteen differing bases between the present set of the subtype B and subtype D 5′UTR region (Figure 3A). The region responsible for attenuated packaging ability on the genetic backbone of subtype B was mapped to a minuscule two-base substitution downstream of PBS. It was rather intriguing that the two consecutive bases (GA to AC) were identified to be essential in inducing the subtype D psi phenotype with subtype B psi (Figure 3D, see NLAINh#8, #8-2, -3, -4). There was no difference in packaging ability when only one base was substituted (Figure 3D, see #8-1 and #8-8).

In the GenBank, the nucleotide base at position 226 of ELI strain is registered as A instead of G (Figure 3, red asterisk). We assume that the inconsistency was originated from a virus propagation in the cells. To prepare the ELI psi segment for the recombinant construction (Figure 1), we propagated the ELI virus in the human primary mononuclear blood cells (PBMCs), followed by the extraction of extrachromosomal DNA (Hirt DNA), PCR amplification, and cloning of the psi fragments [9]. We noticed by sequencing that all of the cloned psi segments from the replication-competent viruses had three bases not identical with those in GenBank, including substitution at position 226 (Figure 3A, red asterisks). The data suggested clonal amplification of a pre-existing psi segment possessing the set of substitutions. Thus, the substitutions are likely to be the ones introduced during the HIV-1 replication rather than PCR amplification. This possibility is supported by the characteristics of the HIV-1 reverse transcriptase; this protein is a highly error-prone polymerase causing a mutation rate up to about 4.1 × 10^-3^ per base per cell [5,6]. Whatever the origin of the substitutions, however, what is clear in this study is that the 226/227 dinucleotide alone is sufficient to attenuate packaging activity of the NL4-3 psi segment (Figure 3D), as seen in the subtype D ELI psi segment (Figure 1).

The finding is in line with the previous study suggesting important roles of unpaired guanosines around the *tandem three-way junction* structure [11]. In that study, Keane et al. [11] showed that substitutions of a set of unpaired discontinuous guanosines (G226A/G292A/G294A/G224), including one at position 226 identified in this study, reduced NC-binding and packaging efficiency. Collectively, these studies suggest that these nucleotide substitutions had impacts on the psi 3D structure for genome packaging.

Indeed, our MD simulations predicted remarkable structural impacts on the psi structure; the 226/227 dinucleotide substitution on the subtype B psi induced notable changes in structural dynamics, fold, and hydrogen-bond networks of the SL2 element, a psi region that is competent for binding to the HIV-1 NC protein [18], with minimal effects on the SL1 element involved in the genome dimerization [27,28,29] (Figure 5, Figure 6 and Figure 7). Increasing evidence indicates that the structural dynamics, fold, and hydrogen-bond networks of biomolecules play key roles in their structural stability and intermolecular interactions [13,14,15,16,17,30,31,32]. Therefore, it is probable that these changes of the psi influence its binding to NC protein, a process involving conformational changes and induced-fit, and consequently affect viral genome packaging efficiency. Together with our experimental data, the present study disclosed a key cis-acting regulator to control the structure and activity of the HIV-1 subtype B psi segment.

In contrast to the effects on the NC-binding region, the effects on the genome dimerization region were minimal, if not at all. The most important region for genome dimerization of the HIV-1 is regarded as SL1, containing the dimerization initiation site [1]. Because SL1 sequences of both B and D clones were completely identical, this result seemed to be reasonable. In fact, our in silico studies failed to detect obvious changes in the structural dynamics, fold, and hydrogen-bond networks of the SL1 element through the dinucleotide substitution (Figure 5, Figure 6 and Figure 7). Thus, the conservation in the structural properties of SL1 due to sequence identity is likely to assure the maintenance of subtype D psi-mediated genome dimerization activities. As genome dimerization is supposed to be a prerequisite for genome packaging [1], present results indicate that the genome packaging attenuation of the virus with subtype D psi RNA occurs outside of the dimerization step within whole packaging processes.

Interestingly, the 227 residues exhibited high levels of variation within and among HIV-1 subtypes reported in the fields (Figure 8). This implies that the residue is mutable during the circulation of HIV-1 in humans. In contrast, the 226 residue was highly conserved in nature, indicating strong functional and/or structural constraints against changes. Taking into consideration data that the effects of double substitutions at positions 226 and 227 are moderate rather than lethal for the genome packaging (Figure 3D) and that the dinucleotide pair rather than the single nucleotide plays pivotal roles in deforming the dynamics, the fold, and the hydrogen-bond networks (Figure 5, Figure 6 and Figure 7), the sequence diversity data raise a possibility that the 226/227 dinucleotide pair together plays a role in the HIV-1 adaptive evolution; it can confer on virus structural flexibility and changeability of the psi for the balanced coordination of genome dimerization and packaging, and/or the best-fit packaging efficiency with the highly variable HIV-1 RNAs and proteins. Further study is necessary to address these issues. Consistently, 226A is always found in association with 227C in the HIV-1 subtype B in the public HIV sequence database [34].

In summary, our study here uncovered the unique biological and structural roles of a dinucleotide sequence within the HIV-1 genomic RNA. These data suggest possible structural mechanisms by which HIV-1 controls genome packaging efficiency without touching genome dimerization efficiency. The data further disclose intramolecular hydrogen-bond networks to control the structure and activity of a functional RNA genome segment. To our knowledge, this is the first report providing a structural basis for the changeability of the HIV-1 genome RNA in response to nucleotide mutations. The obtained information will accelerate our understanding of the regulation of the psi-NC interactions, viral genome packaging, structure-function relationship of distinct psi sequences, and HIV-1 replication and evolution.

## 4. Materials and Methods

**Constructs.** The replication-competent HIV-1 proviral clone pNL4-3 (subtype B) [10], its *Env* mutant pNLNh [37], or its derivative NLAINh [9] were used as progenitors for the mutant constructs described below. Mutant plasmids were constructed with standard molecular biology methods.

**DNA transfection.** 293T cells [38] (approximately 3 × 10^6^) were seeded on dishes (diameter, 100mm) the day before transfection with plasmid DNA (5µg) by means of the calcium phosphate precipitation method [39]. The day after transfection, the supernatant was replaced with a fresh medium.

**Isolation of RNA from virions and cells.** At 48-72 h post-transfection, virus particles were collected from the media as described elsewhere [40]. The physical virus titer was determined with an ELISA assay kit for quantitation of CA-p24 (ZeptoMetrix, Inc., Buffalo, NY). To isolate RNA from particles, virions were disrupted by the addition of 1% sodium dodecyl sulfate (SDS) and treated with proteinase K (300 µg/mL) at room temperature for 60 min, followed by TE-saturated phenol/chloroform extraction, chloroform extraction, and ethanol precipitation.

**Northern blotting analysis.** Pelleted RNA was resuspended in T-buffer (10 mM Tris-HCl-pH 7.5, 1 mM EDTA, 1% SDS, 100 mM NaCl, and 10% formamide), and the thermostability of dimeric viral RNA was determined by incubating RNA aliquots for 10 min at the prescribed temperatures [41]. RNA electrophoresis on native agarose gel and northern hybridization analysis was performed as described elsewhere [37]. Plasmid T7pol [41] was used to synthesize a complementary RNA probe for northern hybridization. In experiments designed to assess the conversion of dimers to monomers, relative amounts of both RNA species were quantitated by phosphorimager analysis (Fujifilm Co., Tokyo, Japan) to determine ratios of dimers and monomers.

**Real-time RT-PCR-based packaging assay.** To accurately quantify genome packaging efficiencies of HIV-1s, a real-time RT-PCR-based packaging assay was developed as described previously [42]. To differentiate the control viral genome from those of the mutants of interest, a series of silent mutations for eight amino acids within the CA region of pNLAINh plasmid [43] was introduced and named as pNLAINh-CAmut. A series of Subtype B-Subtype D chimeric mutants, the descendants of NLAINh, were each separately co-transfected into 293T cells along with pNLAINh-CAmut as a control viral expression construct. Viral and cytoplasmic RNA isolated from the transfectant were applied for real-time RT–PCR with the primers and the probes. By comparing the amount of the control and the mutant RNA in virus and cell, it was possible to determine the effect of each of the mutations on relative encapsidation efficiency. Relative encapsidation efficiencies were calculated as the ratio of the amount of mutant to control (pNLNh-CAmut) genomic RNAs in the virions, with normalization to the cytoplasmic levels of the two RNAs.

**Molecular modeling of the HIV-1 psi RNA.** The reported solution NMR structure [11] (PDB code: 2N1Q) was used for the modeling of the psi region of the HIV-1 NL4-3 clone [10]. The NMR structure covers a minimal region, termed Core Encapsidation Signal Ψ^CES^, that can direct HIV-1 genome packaging [12]. The nucleotide sequence of the NL4-3 Ψ^CES^ is identical to that of the NMR Ψ^CES^ structure [11]. Ψ^CES^ RNAs with nucleotide substitutions were constructed with the “DNA/RNA builder” tool of Molecular Operating Environment “MOE” (Chemical Computing Group Inc., Montreal, Quebec, Canada).

**Molecular dynamics (MD) simulation.** Obtained Ψ^CES^ RNA models were individually subjected to MD simulations under conditions at 1 atm, 310 K, and in 150 mM NaCl. Briefly, the simulations were performed using the pmemd module in the Amber 16 program package [19] with *OL3* force field for the simulation of RNA [44,45]. The TIP3P water model [46] was used for the simulation of an aqueous solution. A non-bonded cutoff of 10 Å was used. Bond lengths involving hydrogen were constrained with SHAKE, a constraint algorithm to satisfy a Newtonian motion [47], and the time for all MD simulations was set to 2 fs. After heating calculations for 20 ps until 310 K using the NVT ensemble, simulations were executed using the NPT ensemble at 1 atm, 310 K, and in 150 mM NaCl for 500 ns.

**Calculation of root means square deviation (RMSD).** The structures at given time points of the MD simulation were superposed with the initial structure, and the RMSDs between the heavy atoms of initial and given structures were calculated using the cpptraj module of AmberTools 16 [19], as described previously [20,21,22,23,24]. In some cases, Ψ^CES^ RNAs were divided into five structural units (U5-AUG stem, PBS stem-loop, SL1, SL2, and SL3), and RMSDs for each unit was individually calculated.

**Analyses of hydrogen-bond networks in RNAs.** To analyze distance-dependent non-covalent intramolecular interactions for RNA folds, hydrogen bonds in the MD-based RNA models were identified and visualized on MOE. The numbers of hydrogen bonds at particular residues were also calculated using the *hbond* command at the cpptraj module. Total 20,000 snapshots in the equilibrium states during 300 and 500 ns of MD simulations were used to calculate the number of hydrogen bonds. Hydrogen-bond networks in the Ψ^CES^ RNAs were visualized using the Cytoscape software version 3.8.2 [33], a software for integrated models of biomolecular interaction networks.

**Shannon entropy analysis.** Nucleotide variation at each position of the psi segment was analyzed with Shannon entropy as described previously [20,36]. Nucleotide sequences of the psi segments were obtained from the HIV sequence database [34]. Shannon entropy was calculated on the basis of Shannon’s equation [35]:

$$H\left(i\right)=-\underset{x_{i}}{\sum }p\left(x_{i}\right)\log_{2}p\left(x_{i}\right)$$

where H(i), p(x_i_), and i indicate the nucleotide entropy score of individual position, the probability of occurrence of a given nucleotide at the position, and the position’s number, respectively. An H(i) score of zero indicates absolute conservation, whereas a score of 2.0 bits indicates complete randomness.

## Supplementary Materials

The following are available online at https://www.mdpi.com/article/10.3390/ijms22073435/s1. Figure S1: Hydrogen-bond networks of the whole Ψ^CES^ region.

## References

[1] Dubois N., Marquet R., Paillart J.C., Bernacchi S. (2018). Retroviral RNA dimerization: From structure to functions. Front. Microbiol..

[2] Bbosa N., Kaleebu P., Ssemwanga D. (2019). HIV subtype diversity worldwide. Curr. Opin. HIV AIDS.

[3] Hemelaar J., Elangovan R., Yun J., Dickson-Tetteh L., Fleminger I., Kirtley S., Williams B., Gouws-Williams E., Ghys P.D., WHO-UNAIDS Network for HIV Isolation Characterisation (2019). Global and regional molecular epidemiology of HIV-1, 1990–2015: A systematic review, global survey, and trend analysis. Lancet Infect. Dis..

[4] HIV Circulating Recombinant Forms (CRFs). https://www.hiv.lanl.gov/content/sequence/HIV/CRFs/CRFs.html.

[5] Abram M.E., Ferris A.L., Shao W., Alvord W.G., Hughes S.H. (2010). Nature, position, and frequency of mutations made in a single cycle of HIV-1 replication. J. Virol..

[6] Cuevas J.M., Geller R., Garijo R., Lopez-Aldeguer J., Sanjuan R. (2015). Extremely High Mutation Rate of HIV-1 In Vivo. PLoS Biol..

[7] Song H., Giorgi E.E., Ganusov V.V., Cai F., Athreya G., Yoon H., Carja O., Hora B., Hraber P., Romero-Severson E. (2018). Tracking HIV-1 recombination to resolve its contribution to HIV-1 evolution in natural infection. Nat. Commun..

[8] Vuilleumier S., Bonhoeffer S. (2015). Contribution of recombination to the evolutionary history of HIV. Curr. Opin. HIV AIDS.

[9] Sakuragi J., Sakuragi S., Ohishi M., Shioda T. (2010). Direct correlation between genome dimerization and recombination efficiency of HIV-1. Microbes Infect..

[10] Adachi A., Gendelman H.E., Koenig S., Folks T., Willey R., Rabson A., Martin M.A. (1986). Production of acquired immunodeficiency syndrome-associated retrovirus in human and nonhuman cells transfected with an infectious molecular clone. J. Virol..

[11] Keane S.C., Heng X., Lu K., Kharytonchyk S., Ramakrishnan V., Carter G., Barton S., Hosic A., Florwick A., Santos J. (2015). RNA structure. Structure of the HIV-1 RNA packaging signal. Science.

[12] Heng X., Kharytonchyk S., Garcia E.L., Lu K., Divakaruni S.S., LaCotti C., Edme K., Telesnitsky A., Summers M.F. (2012). Identification of a minimal region of the HIV-1 5’-leader required for RNA dimerization, NC binding, and packaging. J. Mol. Biol..

[13] Harkness R.W.t., Mittermaier A.K. (2017). G-quadruplex dynamics. Biochim. Biophys. Acta Proteins Proteom..

[14] Karplus M., Kuriyan J. (2005). Molecular dynamics and protein function. Proc. Natl. Acad. Sci. USA.

[15] Keul N.D., Oruganty K., Schaper Bergman E.T., Beattie N.R., McDonald W.E., Kadirvelraj R., Gross M.L., Phillips R.S., Harvey S.C., Wood Z.A. (2018). The entropic force generated by intrinsically disordered segments tunes protein function. Nature.

[16] Bai H., Kath J.E., Zorgiebel F.M., Sun M., Ghosh P., Hatfull G.F., Grindley N.D., Marko J.F. (2012). Remote control of DNA-acting enzymes by varying the Brownian dynamics of a distant DNA end. Proc. Natl. Acad. Sci. USA.

[17] Mlynsky V., Bussi G. (2018). Exploring RNA structure and dynamics through enhanced sampling simulations. Curr. Opin. Struct. Biol..

[18] Amarasinghe G.K., De Guzman R.N., Turner R.B., Chancellor K.J., Wu Z.R., Summers M.F. (2000). NMR structure of the HIV-1 nucleocapsid protein bound to stem-loop SL2 of the psi-RNA packaging signal. Implications for genome recognition. J. Mol. Biol..

[19] Case D.A., Betz R.M., Cerutti D.S., Cheatham T.E., Darden T.A., Duke R.E., Giese T.J., Gohlke H., Goetz A.W., Homeyer N. (2016). AMBER.

[20] Koma T., Kotani O., Miyakawa K., Ryo A., Yokoyama M., Doi N., Adachi A., Sato H., Nomaguchi M. (2019). Allosteric regulation of HIV-1 capsid structure for gag assembly, virion production, and viral infectivity by a disordered interdomain linker. J. Virol..

[21] Matsumoto T., Shirakawa K., Yokoyama M., Fukuda H., Sarca A.D., Koyabu S., Yamazaki H., Kazuma Y., Matsui H., Maruyama W. (2019). Protein kinase A inhibits tumor mutator APOBEC3B through phosphorylation. Sci. Rep..

[22] Yokoyama M., Fujisaki S., Shirakura M., Watanabe S., Odagiri T., Ito K., Sato H. (2017). Molecular dynamics simulation of the influenza A(H3N2) hemagglutinin trimer reveals the structural basis for adaptive evolution of the recent epidemic clade 3C.2a. Front. Microbiol..

[23] Yokoyama M., Naganawa S., Yoshimura K., Matsushita S., Sato H. (2012). Structural dynamics of HIV-1 envelope Gp120 outer domain with V3 loop. PLoS ONE.

[24] Yokoyama M., Nomaguchi M., Doi N., Kanda T., Adachi A., Sato H. (2016). In silico analysis of HIV-1 Env-gp120 reveals structural bases for viral adaptation in growth-restrictive cells. Front. Microbiol..

[25] De Guzman R.N., Wu Z.R., Stalling C.C., Pappalardo L., Borer P.N., Summers M.F. (1998). Structure of the HIV-1 nucleocapsid protein bound to the SL3 psi-RNA recognition element. Science.

[26] Ding P., Kharytonchyk S., Waller A., Mbaekwe U., Basappa S., Kuo N., Frank H.M., Quasney C., Kidane A., Swanson C. (2020). Identification of the initial nucleocapsid recognition element in the HIV-1 RNA packaging signal. Proc. Natl. Acad. Sci. USA.

[27] Ulyanov N.B., Mujeeb A., Du Z., Tonelli M., Parslow T.G., James T.L. (2006). NMR structure of the full-length linear dimer of stem-loop-1 RNA in the HIV-1 dimer initiation site. J. Biol. Chem..

[28] Mujeeb A., Clever J.L., Billeci T.M., James T.L., Parslow T.G. (1998). Structure of the dimer initiation complex of HIV-1 genomic RNA. Nat. Struct. Biol..

[29] Zhang K., Keane S.C., Su Z., Irobalieva R.N., Chen M., Van V., Sciandra C.A., Marchant J., Heng X., Schmid M.F. (2018). Structure of the 30 kDa HIV-1 RNA dimerization signal by a hybrid Cryo-EM, NMR, and molecular dynamics approach. Structure.

[30] Shen L.X., Cai Z., Tinoco I. (1995). RNA structure at high resolution. FASEB J..

[31] Klostermeier D., Millar D.P. (2002). Energetics of hydrogen bond networks in RNA: Hydrogen bonds surrounding G+1 and U42 are the major determinants for the tertiary structure stability of the hairpin ribozyme. Biochemistry.

[32] Jucker F.M., Heus H.A., Yip P.F., Moors E.H., Pardi A. (1996). A network of heterogeneous hydrogen bonds in GNRA tetraloops. J. Mol. Biol..

[33] Shannon P., Markiel A., Ozier O., Baliga N.S., Wang J.T., Ramage D., Amin N., Schwikowski B., Ideker T. (2003). Cytoscape: A software environment for integrated models of biomolecular interaction networks. Genome Res..

[34] HIV Sequence Database. https://www.hiv.lanl.gov/content/sequence/HIV/CRFs/CRFs.html.

[35] Shannon C.E. (1997). The mathematical theory of communication. 1963. MD Comput..

[36] Takahata T., Takeda E., Tobiume M., Tokunaga K., Yokoyama M., Huang Y.L., Hasegawa A., Shioda T., Sato H., Kannagi M. (2017). Critical contribution of Tyr15 in the HIV-1 integrase (IN) in facilitating IN assembly and nonenzymatic function through the IN precursor form with reverse transcriptase. J. Virol..

[37] Sakuragi J., Ueda S., Iwamoto A., Shioda T. (2003). Possible role of dimerization in human immunodeficiency virus type 1 genome RNA packaging. J. Virol..

[38] Graham F.L., Smiley J., Russell W.C., Nairn R. (1977). Characteristics of a human cell line transformed by DNA from human adenovirus type 5. J. Gen. Virol..

[39] Aldovini A., Walker B. (1990). Techniques in HIV Research.

[40] McBride M.S., Panganiban A.T. (1996). The human immunodeficiency virus type 1 encapsidation site is a multipartite RNA element composed of functional hairpin structures. J. Virol..

[41] Sakuragi J.I., Panganiban A.T. (1997). Human immunodeficiency virus type 1 RNA outside the primary encapsidation and dimer linkage region affects RNA dimer stability in vivo. J. Virol..

[42] Sakuragi S., Yokoyama M., Shioda T., Sato H., Sakuragi J.I. (2016). SL1 revisited: Functional analysis of the structure and conformation of HIV-1 genome RNA. Retrovirology.

[43] Drosten C., Seifried E., Roth W.K. (2001). TaqMan 5’-nuclease human immunodeficiency virus type 1 PCR assay with phage-packaged competitive internal control for high-throughput blood donor screening. J. Clin. Microbiol..

[44] Perez A., Marchan I., Svozil D., Sponer J., Cheatham T.E., Laughton C.A., Orozco M. (2007). Refinement of the AMBER force field for nucleic acids: Improving the description of alpha/gamma conformers. Biophys. J..

[45] Zgarbova M., Otyepka M., Sponer J., Mladek A., Banas P., Cheatham T.E., Jurecka P. (2011). Refinement of the Cornell. Nucleic acids force field based on reference quantum chemical calculations of glycosidic torsion profiles. J. Chem. Theory Comput..

[46] Jorgensen W.L., Chandrasekhar J., Buckner J.K., Madura J.D. (1986). Computer simulations of organic reactions in solution. Ann. N. Y. Acad. Sci..

[47] Ryckaert J.-P.C., Ciccotti G., Berendsen H.J.C. (1977). Numerical integration of the cartesian equations of motion of a system with constraints: Molecular dynamics of n-alkanes. J. Comput. Phys..

