# Supplementary material for: Identification of a Novel Cis-Acting Regulator of HIV-1 Genome Packaging
Source: Int J Mol Sci. 2021 Mar 26;22(7):3435. doi: 10.3390/ijms22073435 (PMC8036536; doi:10.3390/ijms22073435)
Supplement: Supplementary file 1 [file ijms-22-03435-s001.zip › 4.Figure S1_psi_sato.docx]

**
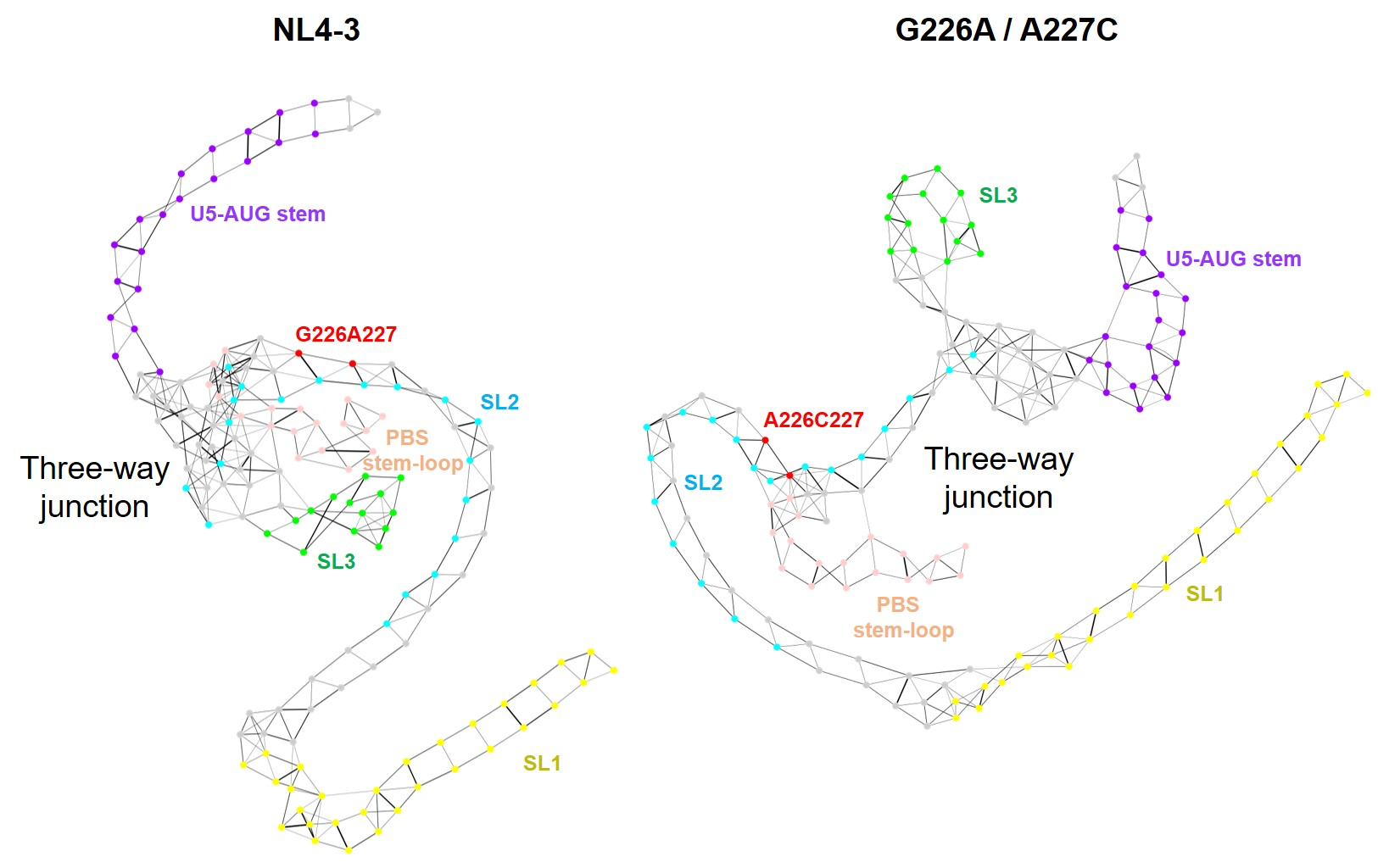
**

**Figure S1. Hydrogen-bond networks of Ψ^CES^ RNAs without or with the 226/227 dinucleotide substitution.** Hydrogen-bonds formed in the Ψ^CES^s without or with the G226A/A227C substitutions were extracted from 20,000 trajectories obtained between 300 and 500 ns after the start of MD simulations as described in the Figure 7 legend and Materials and Methods. The whole hydrogen-bond networks of the Ψ^CES^ RNA structures were visualized by Cytoscape platform [33], where levels of strength of the inter-residue connections were illustrated schematically with thickness of links that directly correlate with the numbers of the hydrogen bonds formed between fluctuated nodes (residues) in psi RNA during MD simulations. Links and edges pf the networks in the structural units of Ψ^CES^, such as U5-AUG stem, SL1, SL2, SL3, and PBS stem-loop, are highlighted with purple, yellow, cyan, green, and skin, respectively.

33. Shannon, P.; Markiel, A.; Ozier, O.; Baliga, N.S.; Wang, J.T.; Ramage, D.; Amin, N.; Schwikowski, B.; Ideker, T. Cytoscape: a software environment for integrated models of biomolecular interaction networks. *Genome Res* **2003**, *13*, 2498-2504, doi:10.1101/gr.1239303.
